# Medical, Dental, and Nursing Students’ Knowledge about Early Childhood Oral Health Care

**DOI:** 10.3390/children6090097

**Published:** 2019-09-02

**Authors:** Wasan Yousef Al-Hatlani, Sanaa Najeh Al-Haj Ali

**Affiliations:** 1 Dental Intern, College of Dentistry, Qassim University, Qassim 51452, Saudi Arabia; 2 Department of Orthodontics and Pediatric Dentistry, College of Dentistry, Qassim University, Qassim 51452, Saudi Arabia

**Keywords:** early childhood oral health care, education, knowledge, medical students, nursing students

## Abstract

Gaps in knowledge of physicians and nurses about early childhood oral health care were reported and are likely due to the poorly focused education on oral health issues; therefore, the purpose of this study was to assess the knowledge level of Qassim University medical, dental and nursing students about early childhood oral health care and its relation to demographic variables, students’ perceived knowledge, satisfaction with their knowledge and interest in further education about the topic. A total of 571 medical, dental, and nursing students received a questionnaire that included demographic questions, questions to assess knowledge level of the students about early childhood oral health care, and questions to assess their perceived knowledge level, satisfaction with their knowledge and interest in further education about the topic. Results of the study revealed that knowledge of dental students was highest (score 7.72 out of 10) followed by nursing students (4.79), and medical students (4.43). Additionally, students with a higher level of perceived knowledge were more likely to score higher. In view of the inadequate knowledge level of medical and nursing students about early childhood oral health care when compared to dental students, improvements in medical and nursing education programs are necessary at Qassim University.

## 1. Introduction

Early childhood caries (ECC) is a significant public health problem which can develop in children as early as ten months of age; therefore, it needs implementation of preventive practices to decrease a child’s risk of developing it [1]. According to the American Academy of Pediatric Dentistry (AAPD), all health professionals are encouraged to participate in preventing ECC by providing services that include initial risk assessment, health promotion, care coordination, oral screening, nutritional counseling, preventive interventions, fluoride varnish application, and referral for dental care [2]. Physicians and nurses have the opportunity to provide oral screening seven times more frequently than dentists for infants and toddlers especially those at high-risk as a result of well child visits [3]. In addition, physicians can identify mothers with high levels of dental caries during obstetrics and gynecology visits, educate them on the importance of their own oral health and the future health of their unborn child, and provide preventive therapies to reduce the incidence of ECC and improve the oral health of their children [4]. 

Similar to the medical home definition that has been adopted by the American Academy of Pediatrics (AAP) in 1992 [5], the AAPD established the dental home concept to reduce the financial burden and decrease the number of dental treatments and procedures experienced by young children and to serve as a higher quality health care alternative in orofacial emergency situations [6]. The dental home may begin in the office of a pediatric dentist and then move to that of a family practitioner, once the child has matured and is more comfortable being treated by the parents’ dentist [7]. Referral by health professionals to a dental home has been recommended based on risk assessment, as early as six months and no later than 12 months of age [8] with subsequent follow up based upon risk assessment. This allows provision of preventive practices and reduces the child’s risk of preventable dental disease [6], which can be extremely important among poor and minority populations who have traditionally had limited access to dental care [3]. 

Various literature reports found gaps in the knowledge of physicians and nurses about early childhood oral health care [9,10,11]. In one study, the majority of pediatricians and general dentists were not advising patients to see a dentist by one year of age, which points out to the need for increased education about the topic in the medical and even dental communities [12]. 

Traditionally, training of physicians and nurses in oral health has been found to be limited [11,13,14,15], which is most likely related to the poorly focused education on oral health issues [16]. One study found that integration of an ECC prevention program into the clinical medical education curriculum resulted in dental caries becoming the eleventh most common diagnosis seen in the clinic, when previously it did not appear in the top 40 [17]. 

The purpose of this study was two-fold: first to assess the knowledge level of Qassim University medical, dental and nursing students about early childhood oral health care; and second to assess its relation to demographic variables, students’ perceived knowledge about early childhood oral health care, their satisfaction with their perceived knowledge and their interest in further education about the topic.

## 2. Materials and Methods

### 2.1. Study Sample

All students from Qassim University college of medicine, college of dentistry, and college of nursing were invited to participate in this cross-sectional study (*n* = 571 total). The surveyed students included the final two clinical years from each college; fourth and fifth year medical students (*n* = 319), fourth and fifth year dental students (*n* = 133), and third and fourth year nursing students (*n* = 119). 

Each student was sent through email or was directly handed a questionnaire and a cover letter explaining the purpose of the study in the period December 2018–April 2019. This study was approved by the ethical committee of college of dentistry-Qassim University (reference number: EA/6011/2018).

### 2.2. Data Collection

Each student was requested to answer a three-part questionnaire that was a modified version from that used in previous reports [13,18], to suit all participating students. Part one of the questionnaire comprised demographic questions including student’s age, gender, college, curriculum inclusion of information about oral health care for young children and awareness about the dental home. Part two of the questionnaire comprised ten questions to assess the actual knowledge level of the students about early childhood oral health care, and part 3 included questions to assess students’ perceived knowledge level, their satisfaction with their perceived knowledge and their interest in further education about the topic. The data used to support the findings of this study are available from the corresponding author upon request.

### 2.3. Data Analyses

Data was analyzed using the SPSS computer software (SPSS Version 20, Chicago, IL, USA). Simple frequency distributions and percentages of the students’ responses toward each question were produced. Frequency responses of part two and three questions were compared using a chi-square test. Students were given a knowledge score which ranged from 0 to 10 according to their answers on part two of the questionnaire. Each correct answer was given one point. Mean knowledge score was calculated for students from different colleges and compared using one-way ANOVA. Ordinal logistic regression analysis was used to identify the association of students’ overall knowledge score with the tested variables. The significance level was *p* < 0.05.

## 3. Results

Table 1 is an overview of students’ demographic data and the response rates. The overall response rate was 71.1%. Percentage of respondents was greatest for fourth year dental students (57.8%) and fifth year medical students (56%). Participation from female students accounted for 56.4% of the total sample. Almost all dental students (92.8%) stated that their curriculum included information about oral health care for young children compared to 43.7% of nursing students, while 57.5% of medical students denied having received such information. Only 17% of the whole sample knew what the dental home is with the lowest percentage recorded for medical students (9.5%).

In part two of the questionnaire, statistically significant differences existed between students from different colleges in all of the questions (*p* < 0.001) (Table 2). The mean knowledge score was highest for dental students 7.72 (out of 10) followed by nursing students (4.79) and medical students (4.43) with statistically significant difference (*p* < 0.001). Seven questions were answered correctly by more than 70% of dental students, while 44–62% of them correctly answered the questions regarding timing of weaning as per AAP and AAPD recommendations, fluoride use for young children, and role of xylitol in dental caries. In case of medical students, two questions only were answered correctly by more than 70% of them; these concerned timing of eruption of the first primary tooth and importance of seeing a dentist at an early age, while three questions were answered correctly by 44–63% of them, and only 10–24% of them correctly answered the questions which concerned timing of weaning, timing of first dental visit, possibility of transmission of cariogenic bacteria from mother to child, fluoride use for young children, and role of xylitol in dental caries.

In case of nursing students, one question was answered correctly by more than 70% of them and this concerned the importance of seeing a dentist at an early age, while five questions were answered correctly by 52–62% of them and four questions were answered correctly by only 12–31% of them; these concerned timing of weaning, timing of first dental visit, fluoride use for young children, and role of xylitol in dental caries.

Responses of the students on part three of the questionnaire are shown in Table 3; a statistically significant difference was found between students from different colleges in these questions (*p* < 0.001). The majority (>70%) of dental students perceived their knowledge level as moderate-high while 86.5% and 73.2% of medical and nursing students respectively perceived their knowledge level as none–little. The great majority (>70%) of students from different colleges were interested in further education about the topic.

Table 4 shows the results of ordinal logistic regression analysis; two factors had a statistically significant effect on the actual knowledge of the students; students’ specialty and level of perceived knowledge (*p* < 0.001 and *p* = 0.003); dental students had 55.5× more likelihood to score higher than medical students and 38.5× more likelihood to score higher than nursing students. In addition, students who perceived their knowledge level as high had 5.68× more likelihood to score higher while those with moderate and little perceived knowledge had 4.173× and 3.724× more likelihood to score higher than those who perceived their knowledge level as none.

## 4. Discussion

The results of this study included surprising and expected findings; differences in knowledge between dental, medical, and nursing students were expected due to the differing focus of the three professions and their different curricula. It was expected that dental students would be most knowledgeable about early childhood oral health; however, the knowledge level achieved by nursing and medical students (knowledge score 4.79 and 4.43, respectively) was surprisingly inadequate in this study. Deficiencies in knowledge of medical and nursing students were particularly noted in issues as timing of weaning, timing of first dental visit, possibility of transmission of cariogenic bacteria from mother to child and the role of xylitol in dental caries as relevant questions were answered correctly by < 1/3rd of the students. In addition, nursing students had deficient knowledge of fluoride use in young children. In similar studies carried out in the United States, India, and Saudi Arabia, medical students performed poorly on questions to these effects [13,15,19,20]. Additionally, nursing students’ knowledge about the timing of the first dental visit was reported to be low in the United States [18]. 

The findings from this study reflect lack of information on early childhood oral health in the curricula of colleges of medicine and nursing. This could be true when the finding that 81% of medical students and 56.3% of nursing students either denied having received such information or were unsure if they had such information is also considered. The ideal approach to oral health care for children is early establishment of a dental home [4]. Surprisingly in this study, only 9.5% of medical students and <30% of nursing students were aware about it. These findings necessitate changes in medical and nursing curricula at Qassim University which may include adding a course on the topic of oral health for young children. Such course can be online as a virtual class, and it can also be shared between dental, medical and nursing students as interdisciplinary education between these three education programs should be the model of future education for pediatric health professionals, which can play an important role in improving oral health in children and referring children at high caries risk, especially those who do not have a dental home but have an established medical one [18]. Encouragingly in this study, the majority of the students were interested to learn more about early childhood oral health care.

When the influence of different variables was assessed on knowledge of the students in this study, other than students belonged to which college, students’ level of perceived knowledge had a significant impact on their actual knowledge in a way that the higher the students perceived their knowledge level, the higher they scored. Therefore, students had somehow accurate perceived knowledge level assessment. Blanch-Hartigan found that medical students more accurately self- assess their knowledge on knowledge-based performance measures when compared to communication-based, standardized patient encounters [21]. Accurate self-assessment of performance is important as it allows students to understand their own strengths and weaknesses and know which areas to focus on [22].

It is important to address the limitations of this study as satisfactory response rates were achieved because different modes were used to deliver the surveys; primarily, electronically and in-person distribution of paper-based surveys for students who did not respond electronically. Several studies that have compared both methods noted that there are no statistically significant differences in response rates and distribution effectiveness [23,24]. In addition, this study was done in one Saudi institution, hence our results may not be generalized to the full body dental, medical, and nursing students in Saudi Arabia. Furthermore, college of nursing students are only females; therefore, the lack of gender influence on knowledge of the students should be further investigated as a respectable percentage of the study population females were from the college of nursing.

## 5. Conclusions

A significant gap in the knowledge of Qassim University nursing and medical students about early childhood oral health care exists when compared to knowledge of dental students, which is concerning as knowledge in this regard has a significant impact on students’ ability to diagnose ECC, install preventive practices for high risk children and refer them for dental care in the near future. This needs to be addressed and improved in medical and nursing education programs at Qassim University. In addition, nursing and medical students themselves should be further motivated to take the initiative to explore and engage by all means to improve their knowledge regarding oral health care for young children, as well-educated health professionals can eventually educate others and can better contribute to patients and parents’ education.

## Figures and Tables

**Table 1:** Respondents’ characteristics (*n* (%)).

Title		Total	Medical Students	Dental Students	Nursing Students
Response rate		406 (71.1%)	252 (79%)	83 (62.4%)	71 (59.7%)
Gender	Female (%)	229 (56.4)	119 (47.2)	39 (47)	71 (100)
Age	18–22	130 (32)	47 (18.7)	28 (33.7)	55 (77.5)
	23–27	276 (68)	205 (81.3)	55 (66.3)	16 (22.5)
Academic level	Fifth year	176 (43.3)	141 (56)	35 (42.2)	-
	Fourth year	193 (47.6)	111 (44)	47 (57.8)	34 (47.9)
	Third year	37 (9.1)	-	-	37 (52.1)
Curriculum included information about oral health care for young children	Yes	156 (38.4)	48 (19)	77 (92.8)	31 (43.7)
	No	177 (43.6)	145 (57.5)	4 (4.8)	28 (39.4)
	Unsure	73 (18)	59 (23.4)	2 (2.4)	12 (16.9)
Awareness about the dental home	Yes	69 (17)	24 (9.5)	27 (32.5)	18 (25.5)
	No	244 (60.1)	176 (69.8)	32 (38.6)	36 (50.7)
	Unsure	93 (22.9)	52 (20.6)	24 (28.9)	17 (23.9)

**Table 2:** Frequency (percentage) of correct answer in questions of part two of the questionnaire.

Variables	Medical Students	Dental Students	Nursing Students	Total	*p*-Value
On average, at what age does the first primary tooth erupt? *Correct response*: 0–12 months	189 (75)	74 (89.2)	38 (53.5)	301 (74.1)	<0.001
By what age do the AAP and AAPD recommend that a child be weaned off? *Correct response*: At 12 months	49 (19.4)	37 (44.6)	22 (31)	108 (26.6)	<0.001
At what age is the child’s first dental visit recommended? *Correct response*: 6–12 months of age	60 (23.8)	62 (74.7)	18 (25.4)	140 (34.5)	<0.001
Is it important for a child to see a dentist at an early age? *Correct response*: Yes	186 (73.8)	80 (96.4)	54 (76.1)	320 (78.8)	<0.001
Can the bacteria that cause tooth decay be transmitted from mother to child? *Correct response*: Yes	57 (22.6)	77 (92.8)	37 (52.1)	171 (42.1)	<0.001
Fluoride prevents tooth decay by making teeth stronger. *Correct response*: Yes	113 (44.8)	68 (81.9)	44 (62)	225 (55.4)	<0.001
A toothpaste containing fluoride should not be used to brush the teeth of children 3 years of age and younger due to the risk of fluorosis. *Correct response*: No	26 (10.3)	51 (61.4)	16 (22.5)	93 (22.9)	<0.001
It is okay to put infants to bed with a bottle of juice or milk. *Correct response*: No	134 (53.2)	78 (94)	42 (59.2)	254 (62.6)	<0.001
Frequent snacking increases the risk of developing early childhood tooth decay. *Correct response*: Yes	159 (63.1)	76 (91.6)	44 (62)	279 (68.7)	<0.001
Xylitol is a sugar substitute that kills the oral bacteria that cause tooth decay. *Correct response*: Yes	32 (12.7)	42 (50.6)	9 (12.7)	83 (20.4)	<0.001
Mean knowledge score (/10) ± SD	4.43 ± 1.47	7.72 ± 1.26	4.79 ± 1.50	5.17± 1.93	<0.001 *

* Using one way ANOVA, all others are chi square tests.

**Table 3:** Responses to part three of the questionnaire (*n* (%)).

Variables	Medical Students	Dental Students	Nursing Students	Total	*p*-Value
Overall perceived knowledge level					<0.001
None	69 (27.4)	4 (4.8)	12 (16.9)	85 (20.9)	
Little	149 (59.1)	14 (16.9)	40 (56.3)	203 (50)	
Moderate	30 (11.9)	48 (57.8)	16 (22.5)	94 (23.2)	
High	4 (1.6)	17 (20.5)	3 (4.2)	24 (5.9)	
Satisfaction with perceived knowledge level					<0.001
Very low	93 (36.9)	1 (1.2)	19 (26.8)	113 (27.8)	
Low	105 (41.7)	18 (21.7)	23 (32.4)	146 (36)	
Moderate	51 (20.2)	50 (60.2)	25 (35.2)	126 (31)	
High	3 (1.2)	14 (16.9)	4 (5.6)	21 (5.2)	
Further education interest on the topic					<0.001
Not interested	78 (31)	13 (15.7)	17 (23.9)	108 (26.6)	
Slightly interested	126 (50)	32 (38.6)	26 (36.6)	184 (45.3)	
Very interested	48 (19)	38 (45.8)	28 (39.4)	114 (28.1)	

**Table 4:** Relation of study variables to knowledge score.

Variables		Mean Knowledge Score	Odds Ratio	95% Wald CI	*p*-Value
Gender (male compared to female (referent))		5.28 vs. 5.08	0.839	0.559–1.262	0.400
Age	18–22	5.18	1	Referent	-
	23–27	5.16	1.248	0.787–1.979	0.347
College	Medicine	4.43	0.018	0.008–0.040	<0.001 *
	Nursing	4.79	0.026	0.010–0.065	<0.001 *
	Dentistry	7.72	1	Referent	-
Academic level	Third year	4.77	1.181	0.464–3.008	0.727
	Fourth year	5.26	1.124	0.746–1.696	0.576
	Fifth year	5.15	1	Referent	-
Curriculum included information about the topic	Yes	6.29	1	Referent	-
	No	4.50	0.894	0.531–1.506	0.673
	Unsure	4.37	0.645	0.358–1.165	0.146
Awareness about the dental home	Yes	5.93	1	Referent	-
	No	4.81	0.935	0.541–1.616	0.811
	Unsure	5.53	1.404	0.770–2.561	0.269
Perceived knowledge level	High	7.0	5.678	1.84–17.526	0.003 *
	Moderate	6.39	4.173	1.966–8.861	<0.001 *
	Little	4.86	3.724	2.191–6.391	<0.001 *
	None	4.02	1	Referent	-
Satisfaction with perceived knowledge level	High	6.86	0.666	0.212–2.088	0.486
	Moderate	6.0	0.735	0.398–1.357	0.325
	Low	4.76	0.567	0.345–0.932	0.06
	Very low	4.45	1	Referent	-
Further education interest on the topic	Not interested	4.73	1	Referent	-
	Slightly interested	4.98	0.989	0.642–1.526	0.962
	Very interested	5.88	1.342	0.807–2.231	0.258

* statistically significant.
